# The Proposed State Board of Medical Examiners

**Published:** 1883-05

**Authors:** 


### Editorial Correspondence.

---

### The Proposed State Board of Medical Examiners.

*To the Editors of the Buffalo Medical and Surgical Journal.*

I have read with a lively interest your vigorous editorial in the March number of the Journal entitled, "The State Board of Medical Examiners."

It seems to me quite certain, that through the dust raised by the discussion of the New Code in the Medical Society of the State of New York, you have discerned a more important question in the report of the Committee on Legislation. Whatever the *status* of the profession of to-day in this country may be, as compared with that of any previous period of its history, it is now evident that its ranks are crowded with half-educated and incompetent men, whose numbers are anually increased by the flood poured on them by the medical schools. This is the fact; why should it be ignored, disguised or excused? The sooner it is recognized and remedied the better for the profession and the people.

Dr. Harvey Jewett, of Canandaigua, in his recent address as President of the Medical Society of the State of New York, said: (San. Eng., Vol. 7, No. 16.) "So long as men are permitted to graduate from some of our most reputable medical schools, to enter the ranks of the regular profession so poorly equipped for life service in a profession which demands the very best talents and preparation, we cannot wonder that quackery continues to flourish;" and again: "The demand is for more thoroughly educated medical men, and that demand must be met by men taken not directly from the farm and the work-shop and the common school, but by those favored by the rigid mental discipline of academy or college, and well grounded in the principles of natural science."

The late Dr. W. W. Green in a presidential address delivered before the Maine Medical Association, said: (*N. Y. Med. Rec.*, Vol. 20, No. 12.) "It is a hackneyed saying with which too many ears are tickled, 'that there is always room for good men.' Applied to the present condition of the profession it is false. Were only good men and the best men admitted, it would undoubtedly be true, but all over the land, in city and country, are well educated, cultured gentlemen, honest and loyal, striving in vain to secure a competence—yes, a bare living even —and too often is disappointment mingled with shame and mortification at the success of ignorant and unprincipled rivals."

It is not denied that capable men are made under the present system. We know that they are. The fault of it lies in the large number of incapables produced. When a young graduate comes, diploma in hand, before a board of examiners who examine him and not his diploma, he often, very often, learns, to his surprise and chagrin, that he must go home and learn the rudiments of the medical sciences which he believed he had mastered. And he does well, if in the same time he gave to "lectures," he surveys the vastness of his ignorance, and beginning at the bottom he builds up a sound foundation for a knowledge of his profession.

Of candidates for appointment in the medical department of one branch of the public service, more than eighty per cent. fail to meet the requirements of the examining board.

A young and accomplished physician of New York City informed me that there is a demand in the country for these men, (i. e., partly qualified men). I replied, that if there were such a demand it should be denied, both in the interest of the public and the profession.

It appears idle to look to the schools for any general reform in their methods. They will continue to furnish cheap diplomas as long as there is a demand for them. This is in accordance with the true commercial principles upon which they are generally founded. The reform movement must be begun and carried on by the profession as a body. Let us have laws creating State Boards of Examiners which shall place a valuation on the unknown quantity of knowledge represented by a diploma. Let the Boards establish a minimum standard of qualifications, which shall represent that reasonable degree of knowledge and skill defined by the common law, and which, heretofore, it has been so difficult to establish as a matter of fact. And so by endeavor to make the profession learned, we shall at least secure that it be competent.

KAPPA.

